# Life history characteristics of a cave isopod (*Mesoniscusgraniger* Friv.)

**DOI:** 10.3897/zookeys.801.23391

**Published:** 2018-12-03

**Authors:** Dávid Deerbák, László Dányi, Elisabeth Hornung

**Affiliations:** 1 Department of Ecology, Institute for Biology, University of Veterinary Medicine Budapest, H-1077 Budapest, Rottenbiller str. 50, Hungary University of Veterinary Medicine Budapest Budapest Hungary; 2 Hungarian Natural History Museum, Department of Zoology, Baross u. 13, H–1088 Budapest, Hungary Hungarian Natural History Museum Budapest Hungary

**Keywords:** continuous breeding, large offspring, K-strategy, small clutch size, sexual size dimorphism

## Abstract

The special environmental conditions of caves provide habitat for several endemic and relict species, among them terrestrial isopods. The Baradla Cave system (north-eastern Hungary) hosts *Mesoniscusgraniger* (Frivaldszky, 1865) (Oniscidea, Microcheta, Mesoniscidae), a pygmy, blind, fragile troglophile woodlice species. Its stable environment can be characterised by the lack of light, high relative humidity (96%), low and constant temperature (about 10 °C). We explored the population characteristics (sex ratio, size distribution) and life history traits of the species (e.g. longevity, reproductive strategy, offspring number, and size). Sex ratio and size distribution of the individuals (head-width measurements) were estimated based on a yearly pooled pitfall-trap data set (N = 677). We studied the species’ reproductive strategy under natural conditions (Baradla Cave, Aggtelek National Park). Model populations were set up in the cave and checked monthly between March and October, 2016 (15 replicates, each with 12 randomly chosen adult individuals; ΣN = 180). Digital photos were taken of the live animals and their length was estimated based on the photos by using ImageJ software (average body length: 6.56 ± 0.79 mm). The results showed female dominance in the population [(male:female = 0.43:0.57); p < 0.001 (GLM)]. Female head width (0.87 ± 0.18 mm) was significantly greater than that of males [0.79 ± 0.08 mm; p < 0.001 (t-test)]. Based on our present data we assume that the offspring number per single female is low (3–5), and new-borns have a relatively large size (body length: 4.22 ± 0.53 mm) compared to the adults. The probability of reproduction was continuous by monthly intervals (binomial test) and longevity exceeds one year. Our results suggest that the species follows a stenodynamic life history.

## Introduction

Life history strategies in terrestrial isopods were first reviewed and divided into stenodynamic and eurodynamic traits by Sutton et al. (1984). These strategies can be matched to endogeic that is soil-active (stenodynamic) and epigeic or surface-active (eurodynamic) species by their characters. A stenodynamic species shows low growth rate, long time to maturity, and produces few and relatively large offspring, while the eurodynamic species have an opposite character. They grow faster, mature earlier, reproduce more frequently, and produce more, but smaller offspring (Achouri et al. 2008, Sutton et al. 1984, Warburg 1994, Brody and Lawlor 1984, Warburg and Cohen 1991, Grundy and Sutton 1989). These strategies are similar to r-K life history strategies (Stearns 1977): stenodynamic species tend to be ‘K’, while eurodynamic species can be best compared to the ‘r’ life history strategy. In reproduction terrestrial isopods show mainly uni-, bi-, or multivoltine iteroparity, sparsely semelparity, and some species are parthenogenetic (Hornung 2011).

The known number of troglobiotic, troglophile terrestrial isopods is over 330 and is increasing as a result of intensive cave faunal surveys worldwide (e.g. Campos-Filho and Araujo 2011, Campos-Filho et al. 2014, 2016, Kováč 2014, Reboleira et al. 2015, Tabacaru and Giurginca 2013, 2014, Taiti 2014, Taiti and Gruber 2008, Taiti and Wynne 2015). The cave environment can be diverse, as abiotic and biotic characteristics depend on parent rock, geological formation, climatic zone, or biome and ecosystem types.

*Mesoniscusgraniger* (Frivaldszky, 1865) was described from Baradla cave, Hungary, and it is one of the two known species of the genus *Mesoniscus* Carl, 1906, Mesoniscidae, Microchaeta (Giurginca 2003, 2005, Gruner and Tabacaru 1963, Schmalfuss 2003). Its known distribution covers the Carpathian Mountains from the Northwest Carpathians (Poland, Slovakia, Hungary), the Western Transylvanian Mountains (Romania, Serbia), the Dinaric Mountains (Serbia, Bosnia and Herzegovina) and the Julian Alps (Slovenia), occurring mainly in caves (Schmalfuss 2003, Giurginca 2003, 2005, Šustr et al. 2005, Piksa and Farkas 2007; Tabacaru and Giurginca 2014). The species was found also endogeic in Poland, Romania, Serbia and Croatia far from caves, in moist and isothermal surface habitats (Mlejnek and Ducháč 2003, Gruner and Tabacaru 1963, J. Bedek, S. Ferenţi, I. Karaman personal communication). Its occurrence is also expected in Ukraine (Šustr et al. 2005). It is the only and abundant terrestrial isopod in the Baradla-Domica cave system in Hungary and Slovakia (Frivaldszky 1865, Šustr et al. 2005). The environmental tolerance and food preference of the species are known from several studies (Gere 1964, Giurginca et al. 2012, Šustr et al. 2005, 2009). The species consumes a mixture of organic and inorganic substrates, e.g. bat guano, rotting wood rests, macroscopic fungi, and algae, with a clear preference for grazing on cave sediment (Giurginca et al. 2012). Its temperature tolerance ranges from -1.5 to 18.5 °C (Gere 1964, Šustr et al. 2005, Šustr et al. 2009, Smrž et al. 2015).

In our study we aimed to define the population characteristics as well as to clarify the life history traits of the species. Our goal was to reveal sex ratio and size distribution of the *M.graniger* population. We aimed to define the average number per female and the average size of offspring. We were particularly interested in the possible ‘trigger’ and any other factors affecting timing of the reproduction of this species under the constant cave conditions, as well as in longevity of the individuals.

## Materials and methods

### Collection of individuals

To estimate sex ratio and size distribution of the population composite pitfall-trap material was used. Traps were placed at 16 plots, in several arms of the Baradla – Domica cave system (in Hungary and Slovakia) in 2012–2013 [Research program: „Management of caves of the World Heritage in the Aggtelek and Slovak Karst“, (HUSK/1101/2.2.1./0180)]. The traps worked through different time periods (mainly four months long). The collected individuals were stored in 70% ethanol.

### Sex and size identification

**Figure 1.**
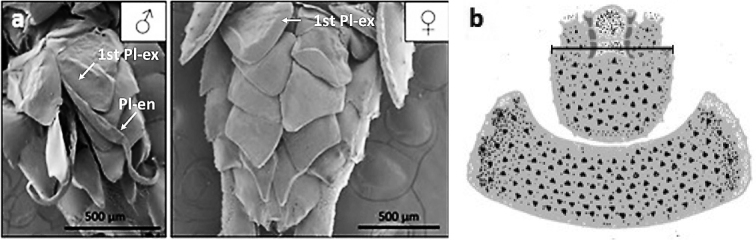
**a** Sexual dimorphism of *Mesoniscusgraniger*. Scale bar 500 µm. Key: 1^st^ Pl-ex – first pleopodite-exopodite, Pl-en – pleopodite-endopodite **b** black line – head-width measurement.

Sex was determined by the presence (♂) or lack (♀) of pleopodite-exopodite extensions (Fig. 1a). The size of individuals was estimated on the basis of head width (Sutton 1968). Digital images were taken under a stereomicroscope (Nikon SMZ800; Nikon E4500 camera) about the head region. The distance between two fixed points of the frontal edge of head capsule was taken as head width (Fig. 1b). The distance was calculated on the images using ImageJ software (https://imagej.nih.gov/ij/). Juvenile category was determined by size smaller than the minimum value of males.

### Model populations

**Figure 2.**
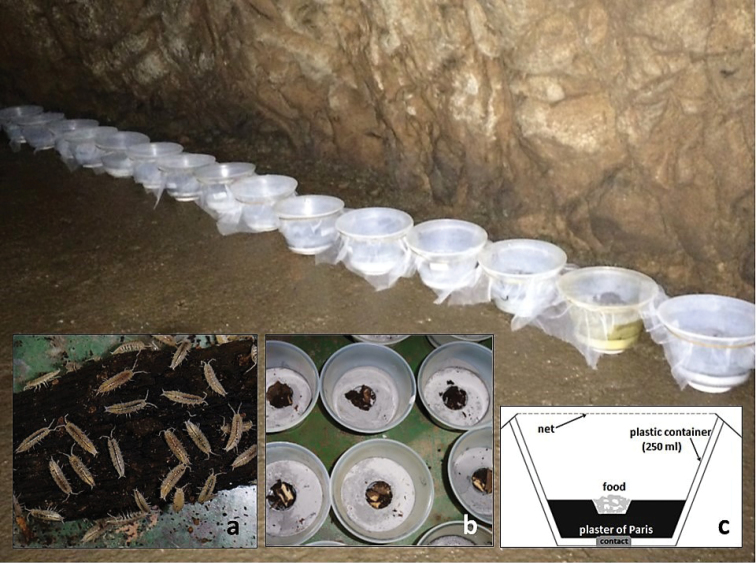
Microcosm model populations in the Baradla cave (north-eastern Hungary). **a***Mesoniscusgraniger* individuals aggregating on decaying wood **b** plan view of a microcosm container with food in the middle **c** schematic cross section of a container.

To explore components of life-history characteristics, such as growth, reproductive events, offspring number, and size, individuals were collected and model populations (N = 15; 12 randomly selected adults in each container; a total of 180 individuals) were set up under natural conditions in a side branch of the Baradla cave (Róka-branch; Aggtelek National Park, Hungary) in March 2016. We used a modified method of Vilisics et al. (2012) to prepare microcosm dishes. Transparent polyethylene containers of 8 cm in height and 9 cm in diameter, without lids were covered by gauze net to ensure ambient humidity but to prevent accidental escape of individuals. The bottom of the containers were filled with a layer of plaster of Paris. Solid plaster has been shown to be suitable in laboratory experiments (e.g., Hassall et al. 1987, van Vliet et al. 1993). We mixed gypsum with activated charcoal powder to detect tiny, depigmented, white animals on a dark background. The bottom of dishes was in contact with the cave floor to ensure the usual wet conditions (Vilisics et al. 2012; Fig. 2).

The source of nutrition was plant debris, bat guano, and loam collected in the cave, placed in small Petri dishes sunk in the plaster of Paris in a central position. Air temperature and relative humidity were measured continuously by a Voltcraft DL-121TH USB data logger. The background factors were constant: 24 hrs dark, temperature 10 °C, and humidity 97%.

### Growth, reproduction, and offspring data

The status and number of individuals and the appearance of new-borns were monitored on a monthly basis (March to October, 2016). The changes were followed at the model populations’ level. In order to minimize external disturbance, we took digital images of each model population with a Nikon D5100 camera. Offspring release could be stated by changes in individual numbers and by size differences (Fig. 4a). The length of individuals (the distance between the medial lobe of the head to the end of the pleotelson) was measured on the photos applying ImageJ software. The camera was placed at a fixed, constant height at each occasion and a calibration scale was added into the container (Fig. 4a).

### Statistics

All statistics were performed with R 3.2.5 RStudio software.

Assuming that the sex ratio for both sexes was 50%, our statistical null hypothesis was that the difference between the two ratios was zero. A binomial test was used to estimate the sex ratio, and the ratios obtained were tested by a generalized linear model (GLM, family = ‘binomial’). The difference between the male and female mean head width was tested by a Welch test.

In the case of each model population temporal changes in the minimum body length values were compared in monthly intervals. Numbers of 0 (no or positive change) or 1 (decreased minimum value) were added depending on results for each microcosm in each consecutive month. We used a 15-element vector for each month based on summed 0 and 1 values. Our statistical null hypothesis was that 50% was the probability of finding smaller sized individuals in any container compared to the previous month. The hypothesis was tested by a binomial test.

The normality of head width and body length variables was tested before the statistical analysis (QQNORM in R 3.2.5).

## Results

### Sex ratio and size distribution

Isopods collected in the pitfall traps were sorted into females, males and juveniles by their sexual characters and size, respectively (Table 1). Based on the binomial test (95% confidence interval) male ratio ranged from 0.39 to 0.47, while that of females from 0.53 to 0.61. Sex ratio was significantly overweighed for females (GLM test, p-value <0.001).

**Figure 3.**
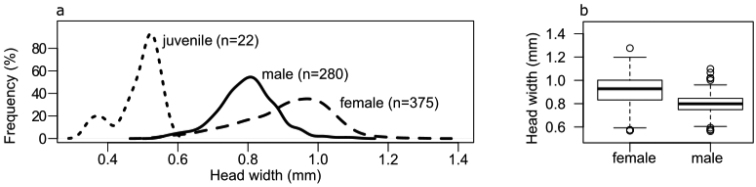
**a** Frequency of size distribution of the studied *Mesoniscusgraniger* population by gender and age **b** Significant differences in head width distribution (average ± SE) of the sexes (Welch test; p-value <0.001).

While females had an average head width of 0.87 mm (SE ± 0.18 mm), the value for males was 0.79 mm (SE ± 0.08 mm) (Fig. 3a, b). Distribution of both male and female head width data was near to normal (Fig. 3). The mean head width of females was significantly higher than that of males (Welch test; p-value < 0.001). There were two markedly separable peaks in the case of juveniles; one between 0.3–0.4 mm and the other at approximately 0.5 mm.

**Table 1.** Distribution of pit-fall trapped individuals by gender and age.

	Males	Females	Juveniles	ΣN
number	280	375	22	677
rate	0.41	0.55	0.04	

### Reproduction events, number, and size of offspring

**Figure 4.**
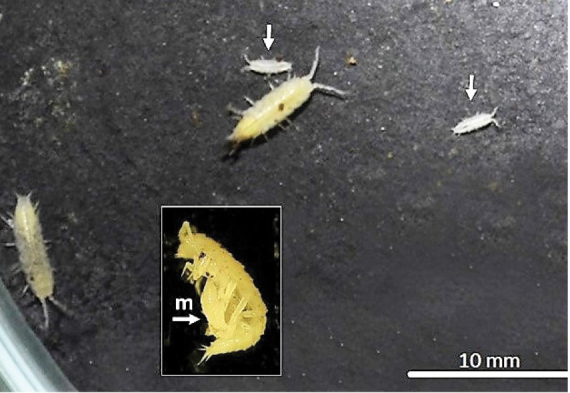
Adults and juveniles (juv – white arrows); insertion: Pregnant female giving birth to a manca (m).

During the study period (8 months) we observed juveniles in 8 containers. Offspring could be identified by their size (Fig. 4). Their number was low: 3–5 individuals per breeding occasion (female) 4.2 ± 0.5 specimens on the average. Mancas were approx. 1/3 of the size of females; average adult size and manca size is 6.65 mm ± 0.68; 4.22 ± 0.53 mm respectively (Fig. 4 insertion). Based on the binomial tests performed, the probability of reproduction, on the model population’s level, was between 30% and 40% in each month.

## Discussion

Assuming that males and females appeared in the traps with equal probability over the sampling period, the sex ratio of *Mesoniscusgraniger* was significantly shifted towards females (♂:♀ = 0.41:0.55). Similarly, the annual distribution of sexes in *Porcellionidespruinosus* (Brandt, 1833) species (♂:♀ = 0.35:0.65) was shifted to females (Achouri et al. 2002). Male to female ratio was found to be 1:5 in *Chetophilosciasicula* Verhoeff, 1908 (Hornung and Szlavecz 2003). Warburg and Cohen (1991) found a fixed male:female ratio of 1:6 in *Schizidiumtiberianum* Verhoeff, 1923, a semelparous species, while Hornung and Warburg (1994) mentioned a constant sex ratio for *Porcellioficulneus* Budde-Lund, 1885, a facultative iteroparous species (♂:♀ = 1:4). In published case studies authors found either equal (1:1) sex ratio (e.g., Carefoot 1973, Tomescu et al. 1992, Warburg and Cohen 1992, Accola et al. 1993, Warburg 2007) or female dominance (Dangerfield and Telford 1994), or sometimes male dominance (Tomescu et al. 1992) for the different species. In iteroparous species females usually prevail over males (Williams and Franks, 1988). The difference in sex ratio might reflect real values characteristic for the population, but in most cases it means a temporal pattern caused by the different surface activity of the sexes (Dangerfield and Hassall 1994). The data of Oberfrank et al. (2011) on *Protracheoniscuspolitus* (C. Koch, 1841) supports the hypothesis of Dangerfield and Hassall (1994): an early male activity was followed by extremely low male presence after onset of reproduction. Hornung et al. (2015) found that male ratio varied over time in the populations of *Trachelipusrathkii* (Brandt, 1833) and *Cylisticusconvexus* (De Geer, 1778) as well. The highest and lowest male : total ratio was found to be 0.57 and 0.26 for *T.rathkii*, 0.61 and 0.27 for *C.convexus*, respectively. This pattern can be explained by the different surface activity of sexes in time: first by the mate search of males and later by the ideal shelter search of gravid females (Dangerfield and Hassall 1994).

Year-round reproduction was reported for *P.pruinosus* (Dangerfield and Telford 1990) and for *Atlantoscianafloridana* (van Name, 1940) (Araujo and Bond-Buckup 2005) under seasonal climate but with relatively favourable microclimate. While *P.pruinosus* was collected in synanthropic habitats which were partially buffered from extreme environmental conditions, *A.floridana* lived in habitats with soil temperature varying from 7.5 to 24.6 °C and litter temperature oscillating between 7.0 and 28.0 °C but little variation in air relative humidity (68.9% to 88.8%). Continuous breeding was also shown in *Porcellioolivieri* Aud. et Sav., and *Agabiformiusobtusus* (Budde-Lund, 1909) (Warburg 1995) under laboratory conditions.

Relative to the adults, few juvenile specimens were collected by the pit-fall traps (Table 1). This might be caused by the selectivity of the method (Topping and Sunderland 1992) and by the low dispersion ability of small juveniles. The bimodal size distribution of immature isopods (Fig. 3a) probably reflects the presence of both mancas (head width between 0.3–0.4 mm) dropped out from the marsupium of trapped pregnant females and larger juveniles (head width 0.5 mm).

Apparent sexual dimorphism in size is not common in terrestrial isopods. One example is in *P.ficulneus* populations where males are significantly smaller in size than females (Hornung, unpubl). Similarly, females were found significantly larger than males both in body length, and in head width in *C.sicula* in a North-American established introduced population (Hornung and Szlavecz 2003).

From our observations we conclude that *Mesoniscusgraniger* is able of continuous reproduction on population level at least from early spring to late autumn, during our study period. Under the constant conditions of the cave environment (10 °C, 97% RH, complete lack of light), reproduction has no abiotic triggers. It is assumed that sexual maturation of females is controlled by their critical mass (Caubet 1998). Female size might be the trigger of reproduction under these conditions. This assumption would be consistent with the constant presence of males but further field studies have to verify this speculation. The low number of offspring per female (in our experiments 3–5), and the large size of the mancas indicate “K” (stenodynamic) life history strategy.

Based on laboratory observations we cannot state but we can assume that the life expectancy of individuals is relatively long, at least 1–1.5 year. By the features found (number of offspring, size of mancas), *M.graniger* can be classified into the stenodynamic life history group together with other endogeic species (Sutton et al. 1984).
